# Impact of type 2 diabetes mellitus on left ventricular diastolic function in patients with essential hypertension: evaluation by volume-time curve of cardiac magnetic resonance

**DOI:** 10.1186/s12933-021-01262-1

**Published:** 2021-03-25

**Authors:** Wei-feng Yan, Yue Gao, Yi Zhang, Ying-kun Guo, Jin Wang, Li Jiang, Yuan Li, Zhi-gang Yang

**Affiliations:** 1 Department of Radiology, West China Hospital, Sichuan University, 37# Guo Xue Xiang, Chengdu, 610041 Sichuan China; 2 Department of Radiology, Key Laboratory of Birth Defects and Related Diseases of Women and Children of Ministry of Education, West China Second University Hospital, Sichuan University, 20# South Renmin Road, Chengdu, Sichuan 610041 P.R. China

**Keywords:** Hypertension, Type 2 diabetes mellitus, Left ventricular diastolic dysfunction, Magnetic resonance imaging, Volume-time curve

## Abstract

**Background:**

Essential hypertension and type 2 diabetes mellitus (T2DM) are two common chronic diseases that often coexist, and both of these diseases can cause heart damage. However, the additive effects of essential hypertension complicated with T2DM on left ventricle (LV) diastolic function have not been fully illustrated. This study aims to investigate whether T2DM affects the diastolic function of the LV in patients with essential hypertension using the volume-time curve from cardiac magnetic resonance (CMR).

**Methods:**

A total of 124 essential hypertension patients, including 48 with T2DM [HTN(T2DM +) group] and 76 without T2DM [HTN(T2DM-) group], and 52 normal controls who underwent CMR scans were included in this study. LV volume-time curve parameters, including the peak ejection rate (PER), time to peak ejection rate (PET), peak filling rate (PFR), time to peak filling rate from end-systole (PFT), PER normalized to end-diastolic volume (PER/EDV), and PFR normalized to EDV (PFR/EDV), were measured and compared among the three groups. Multivariate linear regression analyses were performed to determine the effects of T2DM on LV diastolic dysfunction in patients with hypertension. Pearson correlation was used to analyse the correlation between the volume-time curve and myocardial strain parameters.

**Results:**

PFR and PFR/EDV decreased from the control group, through HTN(T2DM −), to HTN(T2DM +) group. PFT in the HTN(T2DM-) group and HTN(T2DM +) group was significantly longer than that in the control group. The LV remodelling index in the HTN(T2DM −) and HTN(T2DM +) groups was higher than that in the normal control group, but there was no significant difference between the HTN(T2DM −) and HTN(T2DM +) groups. Multiple regression analyses controlling for covariates of systolic blood pressure, age, sex, and heart rate demonstrated that T2DM was independently associated with PFR/EDV (β = 0.252, p < 0.05). The volume-time curve method has good repeatability, and there is a significant correlation between volume-time curve parameters (PER/EDV and PFR/EDV) and myocardial peak strain rate, especially circumferential peak strain rate, which exhibited the highest correlation (r = − 0.756 ~ 0.795).

**Conclusions:**

T2DM exacerbates LV diastolic dysfunction in patients with essential hypertension. The LV filling model changes reflected by the CMR volume-time curve could provide more information for early clinical intervention.

## Background

As two common chronic diseases that threaten human health, essential hypertension and type 2 diabetes mellitus (T2DM) frequently coexist. Approximately two thirds of patients with essential hypertension have impaired glucose tolerance, and hypertensive patients with type 2 diabetes have an increased risk of hospitalization and incidence of adverse cardiovascular events [1, 2]. Left ventricle (LV) diastolic dysfunction is one of the earliest manifestations of myocardial failure and is associated with abnormal blood pressure or glucose metabolism [3, 4]. Therefore, it is of great clinical significance to evaluate the synergistic effect of T2DM and essential hypertension on LV diastolic function. However, the contribution of T2DM to LV diastolic dysfunction in hypertension patients has not been fully elucidated.

Because of its excellent soft tissue resolution and multiplanar, multiparameter imaging, cardiac magnetic resonance (CMR) has become the gold standard for evaluating cardiac structure and function [5, 6]. In previous studies, the volume-time curve parameters obtained by CMR have been considered a promising indicator of LV diastolic function [7, 8] and have been shown to play a role in the diagnosis and evaluation of several cardiovascular diseases [9–12]. To the best of our knowledge, no one has used this method to analyse the additive effects of essential hypertension complicated with T2DM on cardiac diastolic function. Therefore, this study attempted to analyse whether T2DM exacerbates LV diastolic dysfunction in essential hypertensive patients by using CMR volume-time curves.

## Materials and methods

### Study population

This study was approved by the Clinical Trials and Biomedical Ethics Committee of West China Hospital of Sichuan University and carried out following the Declaration of Helsinki (2013 edition). The study subjects were essential hypertension patients who underwent CMR examination in our hospital from May 2016 to October 2020. Hypertension is defined as systolic blood pressure ≥ 140 mmHg and/or diastolic blood pressure ≥ 90 mmHg [13]. The patients with T2DM were screened out from patients with essential hypertension. The diagnosis of T2DM was based on the recommendations of the current guidelines of the American Diabetes Association [14]. The exclusion criteria included heart failure, left ventricular ejection fraction (LVEF) < 50%, coronary heart disease, atrial fibrillation, various congenital heart diseases, valvular heart disease, cardiomyopathy, hyperthyroidism, severe hepatopulmonary dysfunction, secondary hypertension, and estimated glomerular filtration rate (eGFR) < 30 mL/min/1.73 m^2^. In addition, patients with cardiac late gadolinium enhancement (LGE) caused by myocardial infarction or with CMR images of insufficient quality for postprocessing analysis were excluded. Healthy volunteers matched for age and sex with normal blood pressure and blood glucose from our image database were selected as the control group.

Finally, after further exclusion of several patients with unmatched age or sex, 76 essential hypertension patients without T2DM [HTN(T2DM-) group, 39 males and 37 females, mean age 56.43 ± 11.3 years, range 26–78 years], 48 essential hypertension patients with T2DM [HTN(T2DM +) group, 24 males and 24 females, mean age 57.69 ± 12.46 years, range 29–80 years] and 52 healthy controls (26 males and 26 females; mean age 53.29 ± 13.08 years, range 25–76 years) were enrolled in this study. All patients and controls underwent the same CMR examination.

### CMR protocol

CMR was performed using a 3.0 T whole-body scanner (Trio Tim; Siemens Medical Solutions, Erlangen, Germany). All subjects were examined in the supine position. A manufacturer's standard ECG-triggering device and the breath-hold technique were used during the entire examination, and data acquisition was performed during the breath-holding period. Localized imaging, including imaging of the coronal, sagittal, and horizontal planes, was performed by using the True FISP sequence (echo time 1.33 ms, repetition time 710 ms, flip angle 10°, slice thickness 8 mm, space between slices 24 mm, field of view 290 × 373 mm, and matrix size 146 × 224 mm). A balanced steady-state free precession (bSSFP) sequence (field of view [FOV] 250 × 300 mm, repetition time [TR] 39.34 ms, echo time [TE] 1.22 ms, flip angle 40°, slice thickness 8 mm, and matrix size 208 × 139) was used to acquire 8–12 continuous cine images from the mitral valve level to the LV apex in the short-axis view. Vertical LV 2- and 4-chamber long-axis view cine series were acquired as well. LGE images (FOV 400 × 270 mm; TR 750 ms; TE 1.18 ms; flip angle 40°, slice thickness 8 mm) were obtained during the end-diastolic phase 10–15 min after intravenous administration of 0.2 mL/kg gadolinium chelate contrast agent (Gadodiamide, GE Healthcare, Ireland).

### Image analysis

**Fig. 1:**
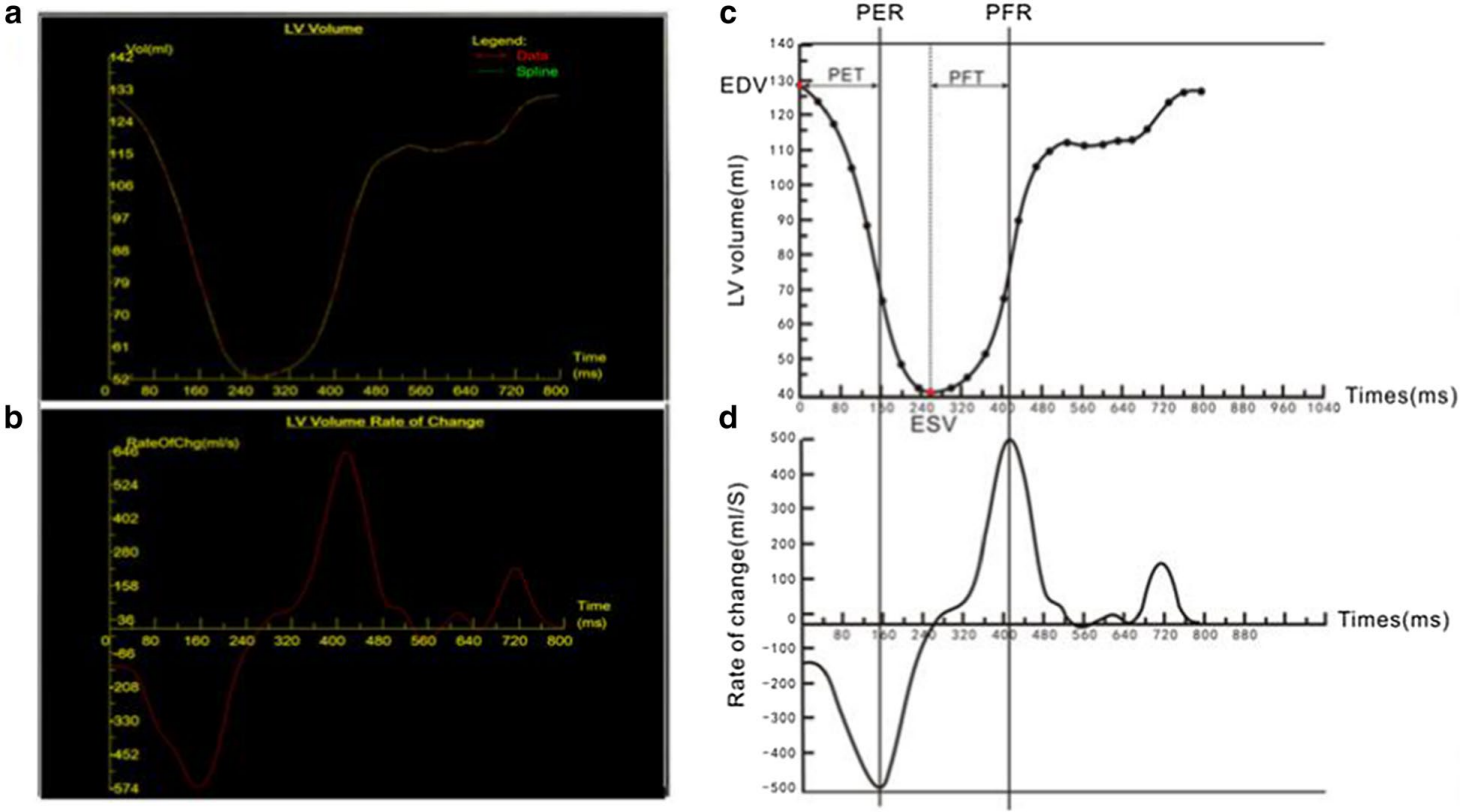
**a**, **b**. Volume-time curves (**a**) and first derivatives (dV/dt) (**b**) depicted by Argus. **c**, **d** The meaning of volume-time curves parameters: peak ejection rate (PER) and peak filling rate (PFR) were determined as peak incremental volume changes, where volume-time curve was steepest, first derivative (dV/dt) was maximal positive or negative

**Fig. 2:**
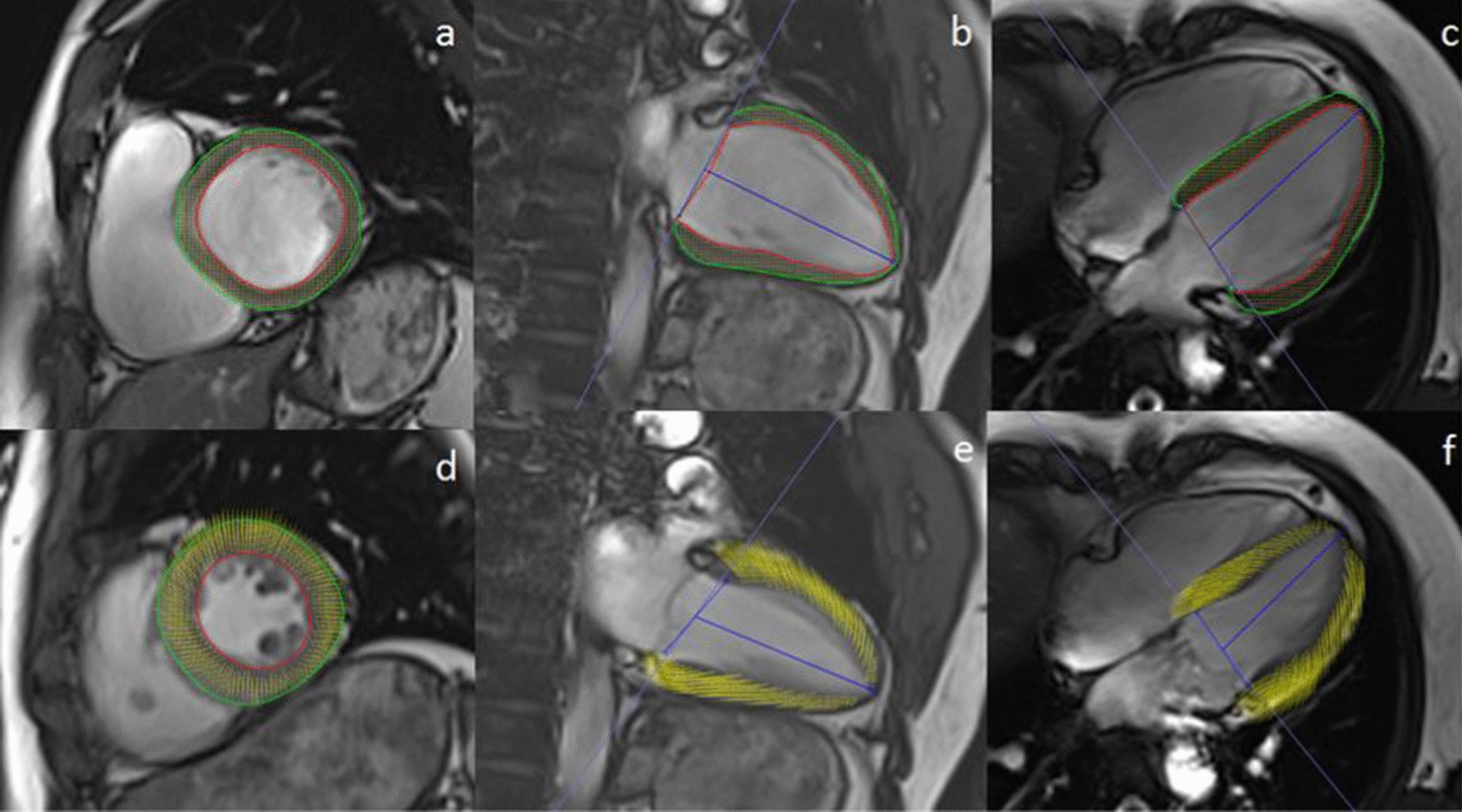
Cardiac magnetic resonance tissue tracking in short-axis and long-axis two-chamber and four-chamber cine images at end-diastole (**a**–**c**) and end-systole (**d**–**f**)

An experienced radiologist analysed the CMR data on an offline workstation without seeing the clinical data and removed patients with LGE. All image postprocessing operations were performed following the latest International Cardiac Magnetic Resonance Association guidelines [15]. The images of eligible subjects were then analysed using offline postprocessing software (Argus, Siemens Medical Solutions, Erlangen, Germany) and offline commercial software (CVI42, v.5.10.2; Circle cardiovascular imaging, Calgary, Canada). Argus was used to draw the endocardial boundary of the LV on each short-axis image, and the volume-time curve parameters, including the peak ejection rate (PER), time to peak ejection rate (PET), peak filling rate (PFR), time to peak filling rate from end-systole (PFT), PER normalized to end-diastolic volume (PER/EDV), and PFR normalized to EDV (PFR/EDV), were obtained. The volume-time curve and the meaning of its parameters are shown in Fig. 1. The end-systolic and end-diastolic endocardium and epicardium on the short axis were drawn by CVI42 to obtain routine cardiac function indexes, including LV EDV, end-systolic volume (ESV), stroke volume (SV), ejection fraction (EF), and LV mass. The LV remodelling index was calculated as LV mass/EDV. At the same time, the end-diastolic endocardium and epicardium of the short axis and two long-axis sections were drawn to analyse the LV strain parameters (Fig. 2), including LV radial global peak strain (GRPS), circumferential global peak strain (GCPS), longitudinal global peak strain (GLPS) and the peak strain rates in these three directions during systole (PSSR) and diastole (PDSR).

### Statistical analysis

Statistical analyses were performed with IBM SPSS (version 22.0, IBM SPSS Inc., Armonk, New York, US). All continuous variables were checked for normality using the Kolmogorov–Smirnov test. Normally distributed data are presented as the mean ± standard deviation, while nonnormally distributed variables are presented as the median and interquartile range. One-way analysis of variance (one-way ANOVA) and Kruskal–Wallis tests were used to compare the baseline characteristics and CMR parameters among the normal, HTN (T2DM-) and HTN(T2DM +) groups. One-way ANOVA was used when the data conformed to the homogeneity of variance and normal distribution assumptions, and the one-way ANOVA was followed by the Student–Newman–Keuls test. Kruskal–Wallis tests were used when the data exhibited skewed distributions. Categorical variables are expressed as frequencies (percentages) and were analysed using the chi-square test. Univariate and multivariate linear regression analyses were used to identify independent correlates of volume-time curve parameters and the basic clinical data. All candidate variables for linear regression analyses were selected based on clinical grounds, and those with a p value < 0.1 in the univariate analysis were included in the stepwise multivariable linear regression analysis. The relationship between the volume-time curve and myocardial strain was analysed by Pearson correlation. Inter- and intra-observer agreements were determined by the evaluation of intraclass correlation coefficients (ICCs). A two-tailed p value < 0.05 was considered significant for all statistical tests.

## Results

### Study population and clinical baseline characteristics

A comparison of basic characteristics among the control, HTN(T2DM-), and HTN(T2DM +) groups is presented in Table 1. Age, sex, body surface area (BSA), plasma triglycerides, high-density lipoprotein cholesterol, and estimated glomerular filtration rate (eGFR) were not significantly different between the observed groups (all p > 0.05), but body mass index (BMI) was higher in both the HTN(T2DM −) and HTN(T2DM +) groups than in the control group (all p < 0.01). There was no significant difference in the course of hypertension between the essential hypertension patients with or without T2DM.

**Table 1:** Basic characteristics of normal controls, essential hypertension patients with diabetes and without diabetes

	Controls	HTN (T2DM-)	HTN (T2DM +)
	n = 52	n = 76	n = 48
Demographics			
Age, years	52.7±13.1	56.4±14.22	57.69±12.46
Female, n (%)	26 (50.0%)	37 (48.7%)	24 (50%)
BMI (kg/m^2^)	22.6±2.9	24.9±2.9*	24.2±2.6*
BSA (m^2^)	1.65±0.18	1.7±0.16	1.68±0.13
Hemodynamic variables			
Systolic blood pressure (mm Hg)	114.8±10.1	143.2±20.7*	140.9±18.3*
Diastolic blood pressure (mm Hg)	73.2±8.6	85.3±16.5*	81.6±10.2*
Laboratory data			
Fasting bloodglucose (mmol/L)	5.21±1.66	5.15±0.7	8.04±3.24*,#
HbA1c, (%)	5.27±0.36	5.58±0.25	6.86±0.97*,#
Plasma triglycerides (mmol/L)	1.35±0.51	1.49±1.08	1.32±0.55
Total cholesterol (mmol/L)	4.71±0.96	4.44±0.89	4.01±1.87
HDL (mmol/L)	1.34±0.33	1.32±0.48	1.34±1.37
LDL (mmol/L)	2.55±0.76	2.53±0.72	2.38±1.11
eGFR (mL/min/1.73 m2)	101.29±16.8	96.86±20.04	90.66±29.39
HTN treatment			
HTN duration (years)	–	6.8±7.9	5.7±5.6
ACEI/ARB, n (%)	–	28 (40)	15 (35)
Beta-blocker, n (%)	–	28 (40)	16 (40)
Calcium channel blocker, n (%)	–	40 (55.7)	20 (47.5)
Diuretics, n (%)	–	11 (15)	6 (15)
Diabetes treatment			
Diabetes duration (years)	–	–	8.22±4.07
Oral, n (%)	–	–	40 (80.2)
Insulin, n (%)	–	–	7 (15.6)

The values are the mean ± SD, Numbers in the brackets are percentages

*HTN* hypertension, *T2DM* type 2 diabetes mellitus, *BMI* body mass index, *BSA* body surface area, *HDL* high-density lipoprotein cholesterol, *LDL* low-density lipoprotein cholesterol, *eGFR *estimated glomerular filtration rate,* ACEI* angiotensin converting enzyme inhibitor, *ARB* angiotensin II receptor blocker

*p < 0.05 versus controls

^#^*P* < 0.05 versus HTN (T2DM-) group

### Comparison of LV function and time‑volume curve parameters

The CMR findings for the observed groups are shown in Table 2. With the exception of the myocardial mass and LV remodelling index in both the HTN(T2DM −) and HTN(T2DM +) groups being significantly higher than that of the control group (all p < 0.001), there was no significant difference in routine CMR parameters among the three groups. GLPS deteriorated from the control group to the HTN(T2DM −) and HTN(T2DM +) groups.

**Table 2:** Comparisons of CMR findings between controls, HTN (T2DM −) group and HTN (T2DM +) group

	Controls	HTN (T2DM−)	HTN (T2DM +)	*P* value
	n = 52	n = 76	n = 48	
CMR-derived cardiac geometric and functional parameters				
Heart rate (beats/min)	69.2 ± 11.3	73.5 ± 14.3	71.82 ± 12.33	0.196
LVEF (%)	70.8 ± 4.9	71.3 ± 8.1	69 ± 9.3	0.326
LVEDVI (ml/m^2^)	68.21 ± 12.09	70.16 ± 14.8	70.24 ± 11.76	0.702
LVESVI (ml/m^2^)	20.33 ± 7.27	20.91 ± 9.56	22.06 ± 8.47	0.626
LVSVI (ml/m^2^)	47.89 ± 6.71	49.25 ± 8.33	48.18 ± 9.25	0.641
LVMASS(g)	83.30 ± 23.23	102.4 ± 33.3*	100.4 ± 27.50*	0.000
Standardized Left Ventricular Mass(g/m^2^)	47.92 ± 12.16	59.7 ± 15.65*	56.6 ± 15.4*	0.000
LV-remodeling index (g/ml)	0.73 ± 0.15	0.83 ± 0.14*	0.89 ± 0.16*	0.000
CMR volume-time curve parameters of left ventricle				
PER (ml/s)	387.48 ± 99.63	417.89 ± 115.86	384.42 ± 92.66	0.088
PET (ms)	109.07 ± 23.84	103.46 ± 29.01	110.76 ± 30.52	0.116
PFR (ml/s)	390.61 ± 84.98	357.8 ± 95.9*	319.8 ± 67.8*^,#^	0.000
PFT (ms)	124.88 ± 24.43	149.14 ± 41.80*	158.42 ± 36.9*	0.000
PER/EDV	3.26 ± 0.62	3.57 ± 0.89	3.49 ± 0.66	0.154
PFR/EDV	3.45 ± 0.55	3.05 ± 0.75*	2.78 ± 0.69*^,#^	0.000
CMR strain parameters of left ventricle				
PS (%)				
Radial	38.89 ± 8.37	37.57 ± 12.22	36.0 ± 10.59	0.552
Circumferential	− 21.1 ± 2.86	− 21.1 ± 2.89	− 20.26 ± 3.68	0.170
Longitudinal	− 15.13 ± 2.64	-13.90 ± 2.83*	− 11.91 ± 3.62*^,#^	0.004
PSSR (1/s)				
Radial	1.94 ± 0.58	2.11 ± 0.79	2.06 ± 0.99	0.822
Circumferential	− 1.01 ± 0.20	− 1.08 ± 0.27	− 0.92 ± 0.50^#^	0.041
Longitudinal	− 0.68 ± 0.34	− 0.81 ± 0.25	− 0.61 ± 0.5	0.232
PDSR (1/s)				
Radial	− 2.62 ± 1.30	− 2.54 ± 1.27	− 2.09 ± 0.90*	0.031
Circumferential	1.30 ± 0.26	1.41 ± 0.38	1.12 ± 0.23*^,#^	0.007
Longitudinal	0.91 ± 0.26	0.96 ± 0.28	0.84 ± 0.33	0.574

*HTN* hypertension, *T2DM* type 2 diabetes mellitus, *LV* left ventricular, *EF* ejection fraction, *EDV* end diastolic volume, *ESV* end systolic volume, *SV* stroke volume, *I* indexed to BSA, *PET* time to peak ejection rate, *PFT* time to peak filling rate from end-systole, *PER* peak ejection rate, *PFR* peak filling rate, *PS* peak strain, *PSSR* peak systolic strain rate, *PDSR* peak diastolic strain rate

*p < 0.05 versus controls;

^#^*p* < 0.05 versus HTN (T2DM-) group

**Fig. 3:**
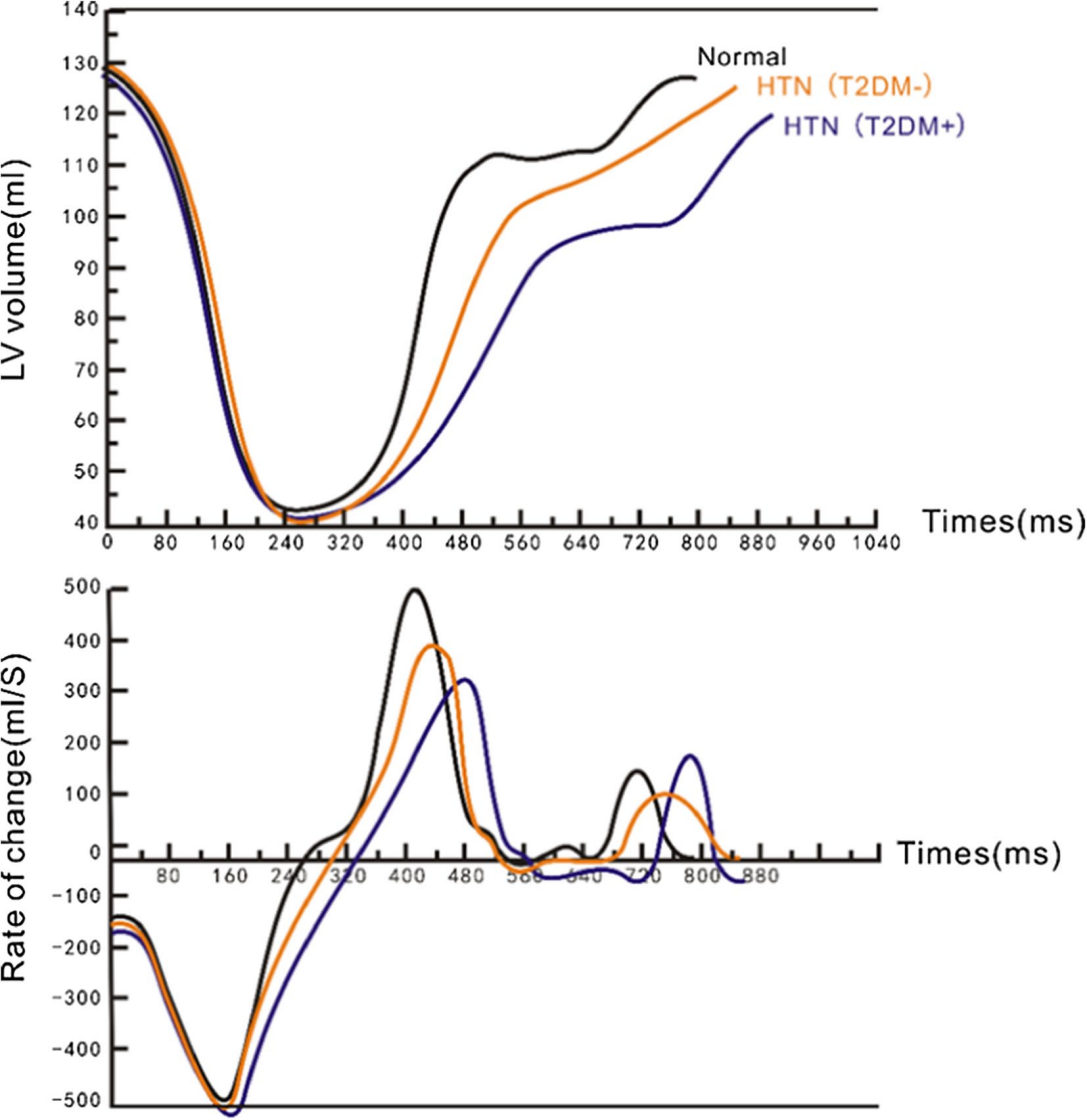
Comparison of LV volume-time curves in healthy participants (normal) and patients. *HTN* hypertension, *T2DM* type 2 diabetes mellitus, *LV* left ventricular

During cardiac systole, there was no significant difference in volume-time curve parameters among the three groups. During diastole, PFT in the HTN(T2DM-) group and the HTN(T2DM +) group were longer than those in the control group, while both the PFR and PFR/EDV values were the lowest in the HTN(T2DM +) group, followed by the HTN(T2DM-) group, with the normal control group having the largest values; all comparisons were statistically significant (all p < 0.05). The typical LV filling patterns of the three groups of subjects are shown in Fig. 3.

### Regression analysis of LV diastolic function parameters in hypertension patients

As shown in Table 3, age, sex, BSA, diabetes, HR, SP, and eGFR were initially screened based on clinical grounds and were assessed using univariable analysis. Univariate linear regression analyses showed that HR had a positive effect on the PFR/EDV (β = 0.027; 95% confidence interval [CI], 0.019 to 0.035; P < 0.001) and that age (β =  − 0.016; 95% CI  − 0.024 to − 0.008; P < 0.001), male sex (β =  − 0.377; 95% CI   − 0.594 to − 0.161; P = 0.01) and diabetes (β =  − 0.429; 95% CI  − 0.669 to − 0.189; P = 0.010) had a negative effect on the PEF/EDV. Multivariable linear regression analyses demonstrated that considering the covariates of age, sex, BSA, and heart rate, T2DM was independently associated with PFR/EDV (β = − 0.186, p = 0.037, model R^2^ = 0.432).

**Table 3:** Univariable and multivariable linear regression analysis of PFR/EDV in all HTN patients (n = 124)

	Univariable		Multivariable		
	β	P value	β	P value	R^2^
Age (y)	− 0.016	0.000	− 0.241	0.001	0.432
Sex (female)	− 0.259	0.01	− 0.234	0.005	
BSA	− 0.087	0.931	–	–	
Diabetes	− 0.186	0.044	− 0.252	0.037	
HR	0.027	0.000	0.410	0.000	
SP	0.018	0.17	–	–	
eGFR	0.144	0.183	–	–	

*HTN* hypertension,* BSA* body surface area, *HR* heart rate. Variables for multivariable model were selected on clinical grounds, guided by univariable correlation with P value < 0.10 and the absence of collinearity

### Correlations between time-volume curve parameters and LV global strain parameters

**Fig. 4:**
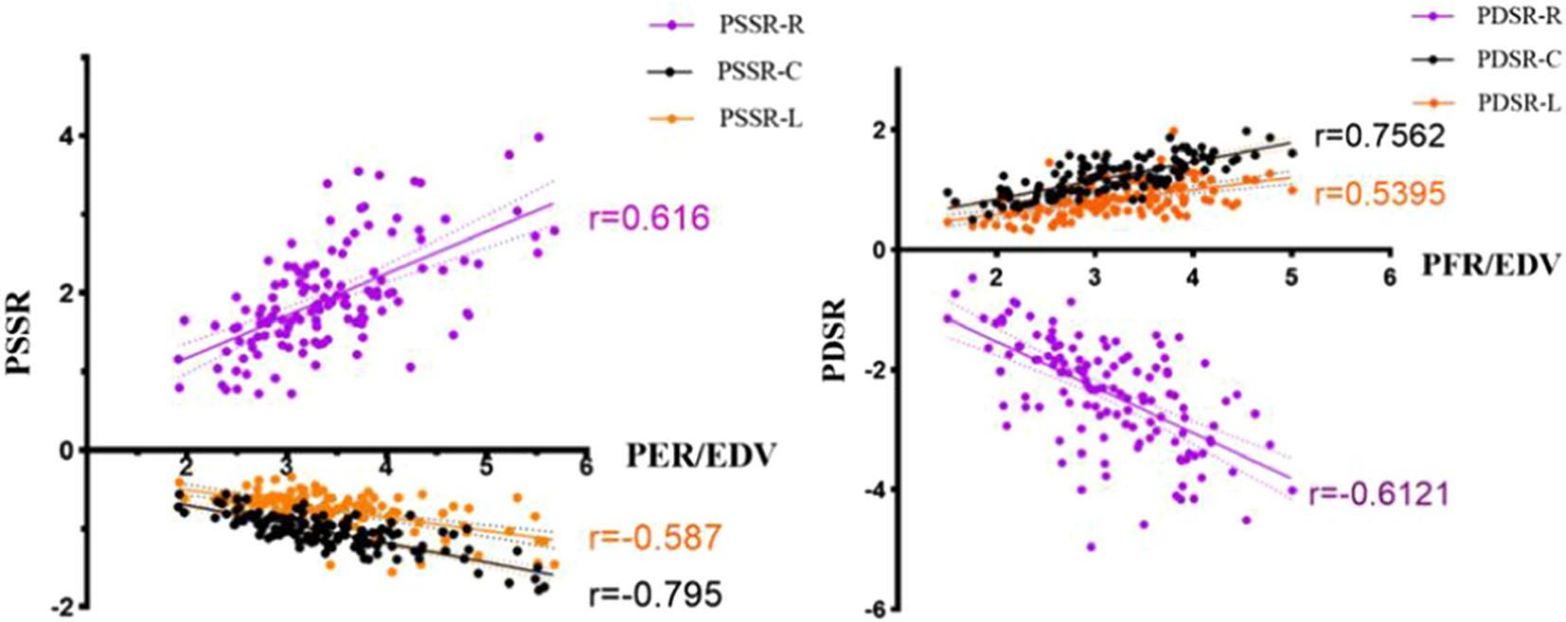
The relationship between peak strain rate in different directions and normalized peak volume change rate. *EDV* end diastolic volume, *PER* peak ejection rate, *PFR* peak filling rate, *PSSR* peak systolic strain rate, *PDSR* peak diastolic strain rate, *R* radial, *C* circumferential, *L* longitudinal

As shown in Table 4 and Fig. 4, significant linear correlations were observed between time-volume curve parameters and LV global strain parameters. In particular, there was a clear association between the circumferential myocardial strain rate and the volume change rate. Circumferential PSSR and circumferential PSDR were significantly correlated with PER/EDV (r =  − 0.795, p < 0.001) and PFR/EDV (r = 0.756, p < 0.001), respectively.

**Table 4:** Correlation analysis of LV global strain parameters with the time-volume curve parameters

	PER		PFR		PER/EDV		PFR/EDV	
	r	P value	r	P value	r	P value	r	P value
PS (%)								
Radial	− 0.012	0.89	0.04	0.648	.414#	0.000	.449#	0.000
Circumferential	− 0.006	0.946	− .219*	0.011	− .391#	0.000	− .588#	0.000
Longitudinal	0.146	0.092	− 0.097	0.267	− .226#	0.009	− .467#	0.000
PSSR (1/s)								
Radial	0.161	0.063	− 0.019	0.829	.603#	0.000	.372#	0.000
Circumferential	− .329#	0.000	− 0.047	0.588	− .795#	0.000	− .407#	0.000
Longitudinal	− .186*	0.031	− 0.087	0.315	− .288#	0.001	− 0.141	0.103
PDSR (1/s)								
Radial	0.084	0.334	− .254#	0.003	− .197*	0.022	− .549#	0.000
Circumferential	− 0.009	0.918	.412#	0.000	.358#	0.000	.756#	0.000
Longitudinal	− 0.11	0.206	0.138	0.113	.302#	0.000	.540#	0.000

Abbreviation of PET, PFT, PER, PFR, PS, PSSR, PDSR, are shown in Table 2

*p < 0.05; #p < 0 .01

### Intra- and interobserver variability

As demonstrated in Table 5, there were excellent intra- and interobserver agreements in the measurement of time-volume curve parameters (ICC = 0.885 ~ 0.937 and 0.831 ~ 0.916, respectively).

**Table 5:** Inter- and intra-observer variability of time-volume curve

	Intra-observer (n = 30)		Inter-observer (n = 30)	
	ICC	95% CI	ICC	95% CI
PET	0.885	0.828–0.943	0.831	0.735–0.899
PFT	0.898	0.865–0.934	0.852	0.796–0.912
PER	0.887	0.873–0.924	0.879	0.852–0.898
PFR	0.937	0.895–0.976	0.916	0.865–0.942

*ICC* intraclass correlation coefficient, *CI* confidence interval; Abbreviation of PET, PFT, PER, PFR are shown in Table 2

All p < 0.01

## Discussion

Hypertension is the most common cardiovascular risk factor involving multiple systems and causes organ and tissue damage. Heart failure is one of the complications most closely related to blood pressure [16]. As a common metabolic disease, T2DM also has adverse effects on the heart and often coexists with essential hypertension [17, 18]. In this study, our main findings are as follows: (1) LV diastolic function was impaired in patients with essential hypertension, even in the absence of clinical evidence of heart failure; (2) T2DM aggravated LV diastolic dysfunction in patients with essential hypertension; and (3) in both systolic and diastolic phases, myocardial circumferential strain had the greatest influence on LV volume.

Based on our results, compared with the respective findings in the normal control group, the PFR and PER/EDV were lower and PFT was significantly prolonged in the HTN(T2DM-) group. These changes represent the deterioration of LV diastolic function in hypertensive patients [19]. Extensive research has shown that when hypertension exists as a single factor, its effect on the heart is mainly reflected in the abnormal accumulation of fibrous collagen and compensatory LV hypertrophy caused by chronically increased LV workload [20]. Once these changes cause myocardial relaxation to slow down, LV diastolic function is impaired [21]. Although patients with an EF < 50% and/or myocardial infarction were strictly excluded, our findings in patients with essential hypertension alone are consistent with findings of previous echocardiography studies [22, 23]. At present, echocardiography is the primary method for the clinical evaluation of cardiac diastolic function, and it has been widely used in scientific research [24, 25]. However, in some special cases, such as obesity, chronic obstructive pulmonary disease and patients with chest pain or recent surgery, echocardiography has limitations. With the advancement of scanning and postprocessing techniques, CMR is expected to become another important imaging method in this field [26, 27].

When patients with essential hypertension are complicated with T2DM, we found that PFR and PFR/EDV were further decreased in HTN(T2DM +) patients compared with the same parameters in HTN(T2DM-) patients. In addition, after considering confounders such as age, sex, and BSA, the adverse effects of T2DM on the LV diastolic function of patients with hypertension remained. Previous studies have shown that due to many individual or common pathophysiological factors, such as myocardial hypertrophy, cardiac steatosis, interstitial fibrosis, and myocardial energetic impairment, an adverse positive feedback cycle exists between hypertension and T2DM [28–31]. Although the interaction between various factors has not been fully elucidated, systemic vascular dysfunction and endocrine-related microcirculation disorders are considered to play essential roles [32, 33]. In the early stages of cardiac damage associated with only diastolic dysfunction, these adverse effects are often difficult to identify using routine cardiac function parameters. Nevertheless, the differences in LV filling patterns shown by the volume-time curve between HTN(T2DM +) and HTN(T2DM-) in this study could intuitively indicate that T2DM further reduces LV compliance in hypertensive patients during passive diastole.

Despite the use of antihypertensive drugs, the LV mass and remodelling index in our patients with essential hypertension were higher than those in the participants in the normal group. This result is consistent with previous studies that indicated that myocardial remodelling independent of antihypertensive effects could occur over a longer time [32, 34]. In addition to cardiac load, several previous studies have shown that abnormal glucose metabolism and insulin resistance also lead to adverse remodelling of the heart [35–37]. However, there was no significant difference in the LV remodelling index between the HTN(T2DM-) group and HTN(T2DM +) group according to our results. This nonsynchronization of function and morphology suggests that T2DM has adverse effects on LV diastolic function in essential hypertension patients, even if the overall cardiac structure has not been further changed.

In recent years, myocardial strain has been considered a reliable index to evaluate cardiac function [38]. By analysing the correlation between the volume-time curve and myocardial strain, we further confirmed that myocardial strain in different directions has different degrees of association with LV volume. Previous investigators have found that circumferential motion contributes twice as much to EF as longitudinal motion [39]. In this study, we analysed the correlation of longitudinal, radial, circumferential peak strain rates of systole and diastole with the peak volume change rates of the corresponding cardiac phase and demonstrated that the LV volume is most related to the circumferential motion of the myocardium in both the systolic and diastolic periods. Other echocardiographic studies have shown that many diseases, including essential hypertension and T2DM, are most likely to cause changes in the subendocardium, which is mainly composed of longitudinally oriented fibres [40–42]. Thus, our results may explain from another point of view why GLPS could detect slight myocardial deterioration, but the diseases that lead to these abnormalities result in no significant reduction in EF.

There are some limitations in our research. First, as a single-centre study, we adhered to strict inclusion and exclusion criteria to limit the influence of confounding factors, which resulted in a reduced sample size. Second, this study confirmed that the volume-time curve is sensitive to subclinical LV diastolic dysfunction, but it is still necessary to further explore its specific application in different clinical diseases. Finally, since this is a retrospective study, the evolution of the diseases needs to be discussed in further follow-up or prospective studies in the future.

## Conclusions

T2DM aggravates the damage of LV diastolic function in essential hypertension patients, even in those without further remodelling of a cardiac structure. The change in LV filling pattern reflected by the CMR volume-time curve could reflect this adverse effect earlier than conventional cardiac function parameters, which may provide more valuable information for clinical treatment.

## Data Availability

The datasets used and analyzed during the current study are available from the corresponding author on reasonable request.

## Abbreviations

HTN: Hypertension
T2DM: Type to diabetes mellitus
LV: Left ventricular
CMR: Cardiac magnetic resonance
EDV: End-diastolic volume
ESV: End-diastolic volume
EF: Left ventricular ejection fraction
SV: Stroke volume
I: Indexed to BSA
PET: Time to peak ejection rate
PFT: Time to peak filling rate from end-systole
PER: Peak ejection rate
PFR: Peak filling rate
PS: Peak Strain
PSSR: Peak Systolic Strain Rate
PDSR: Peak Diastolic Strain Rate
